# The RETRIAD Study: Comparative First-Pass Reperfusion Performance of EmboTrap III, Trevo NXT, and APERIO Stent Retrievers in Acute Large-Vessel Occlusion Stroke: A Single-Center Retrospective Cohort Study

**DOI:** 10.3390/jcm15218412

**Published:** 2026-10-29

**Authors:** Halil İbrahim Akçay

**Affiliations:** 1 Department of Neurology, Faculty of Medicine, Niğde Ömer Halisdemir University, Bor Yolu Üzeri, Central Campus, 51240 Niğde, Türkiye; hiakcay38@gmail.com; 2 Department of Neurology, Niğde Ömer Halisdemir University Training and Research Hospital, Aşağı Kayabaşı Mahallesi, Hastaneler Caddesi, Kumluca Mevkii, 51100 Niğde, Türkiye

**Keywords:** mechanical thrombectomy, first-pass effect, stent retriever, EmboTrap III, Trevo NXT, APERIO, large-vessel occlusion, eTICI

## Abstract

**Background/Objectives:** Contemporary stent retrievers differ in design, yet direct comparative evidence regarding their first-pass reperfusion performance using a standardized endpoint remains limited. This study compared first-pass reperfusion outcomes among APERIO, Trevo NXT, and EmboTrap III. **Methods:** In this exploratory, single-center retrospective cohort study, 200 patients were screened between 1 March 2024 and 1 August 2026. Forty patients had not completed 90-day follow-up at the time of the initial data analysis. An additional 104 patients were excluded because of tandem occlusion (*n* = 29), recanalization after intravenous thrombolysis before thrombectomy (*n* = 8), distal vessel occlusion (*n* = 30), or successful direct aspiration without stent-retriever deployment (*n* = 37). The final cohort comprised 56 patients treated with first-line combined stent-retriever and aspiration thrombectomy using APERIO (*n* = 28), Trevo NXT (*n* = 14), or EmboTrap III (*n* = 14). The primary endpoint was strict first-pass effect (FPE), defined as first-pass expanded Treatment in Cerebral Ischemia (eTICI) grade 2c–3. **Results:** Strict FPE was achieved in 16/28 (57.1%), 9/14 (64.3%), and 9/14 (64.3%) patients, respectively (global exact *p* = 0.879). Both comparisons with APERIO yielded an absolute risk difference of +7.1 percentage points (95% CI, −23.2 to +33.6). Exploratory adjusted analyses yielded imprecise estimates. Heidelberg-classified symptomatic intracranial hemorrhage occurred in zero, three, and two patients, respectively. **Conclusions:** No statistically detectable difference in strict FPE was demonstrated among the three device-based treatment strategies. However, the small sample size, wide confidence intervals, and nonrandomized treatment allocation preclude conclusions regarding device equivalence or causal effects.

## 1. Introduction

Endovascular thrombectomy is established as a standard treatment for acute ischemic stroke caused by large-vessel occlusion (LVO), with randomized-trial meta-analysis demonstrating substantial improvement in functional outcome when timely reperfusion is achieved [1]. As contemporary thrombectomy systems have matured, the procedural question has shifted from whether reperfusion can be achieved to how rapidly, completely, and safely it can be obtained. Final eTICI ≥ 2b reperfusion rates are now high with modern devices, reducing the discriminatory value of final ≥2b reperfusion alone for comparing thrombectomy strategies.

The first-pass effect (FPE) was introduced as a procedural performance metric because achieving substantial reperfusion with a single thrombectomy attempt is associated with better clinical outcomes and avoids the potential injury, distal embolization, and time cost of repeated device passes [2]. Increasing evidence also supports a more stringent angiographic target. A recent meta-analysis of 10,235 patients found that eTICI 2c–3 reperfusion was associated with greater functional independence and lower mortality and symptomatic intracranial hemorrhage than eTICI 2b [3]. Accordingly, first-pass near-complete or complete reperfusion (eTICI 2c–3) provides a clinically relevant endpoint for comparing contemporary thrombectomy devices.

Modern stent retrievers differ in cell architecture, device length and diameter, radiopacity, delivery characteristics, and mechanisms of clot integration. Nevertheless, whether these engineering differences translate into clinically meaningful differences in first-pass reperfusion remains uncertain. In the ROSSETTI registry, stent-retriever brand, diameter, and length were not independently associated with first-pass reperfusion among M1 occlusions treated with a stent retriever and balloon-guide catheter [4]. Similarly, a nationwide MR CLEAN Registry analysis found only small differences in outcomes among commonly used stent retrievers and no significant differences in first-pass reperfusion, although the devices and treatment era largely preceded the current generations evaluated here [5].

Evidence for the three contemporary platforms examined in the present study is derived mainly from device-specific series. Trevo NXT achieved first-pass mTICI ≥ 2c in 54% of 80 patients in a multicenter European cohort [6], whereas a multicenter US EmboTrap III study reported first-pass mTICI ≥ 2c in 53.3% of 92 patients [7]. APERIO Hybrid studies have demonstrated high reperfusion rates in real-world practice, but first-pass definitions have varied across reports, limiting direct comparisons [8,9,10]. Contemporary head-to-head data using a uniform strict first-pass endpoint are therefore limited. Evidence regarding balloon-guide catheter (BGC) use remains mixed. A matched-cohort study reported higher first-pass reperfusion rates and improved neurological outcomes with BGC use [11], whereas a meta-analysis of randomized controlled trials found no significant improvement in 90-day functional independence or final eTICI 2c–3 reperfusion [12]. The study was named RETRIAD (REtriever TRIad Assessment of Devices), reflecting its comparative evaluation of three contemporary stent-retriever platforms. The present study aimed to compare first-line APERIO, Trevo NXT, and EmboTrap III using strict FPE, defined for this analysis as first-pass eTICI 2c–3, as the primary endpoint. We additionally examined how exploratory estimates varied across restricted occlusion-site and BGC-use subgroups; these analyses cannot establish causal device effects.

## 2. Materials and Methods

### 2.1. Study Design and Population

This exploratory single-center retrospective comparative cohort was conducted at Niğde Ömer Halisdemir University Training and Research Hospital, Türkiye. The initial screening frame comprised 200 patients treated between 1 March 2024 and 1 August 2026. Forty patients treated in the later accrual period (1 May–1 August 2026) were excluded because their 90-day follow-up periods had not been completed at the time of the initial data analysis. Among the remaining 160, four additional non-overlapping primary exclusion categories were reconstructed for the flow accounting: tandem occlusion (*n* = 29), spontaneous/IV thrombolysis-associated recanalization documented at angiography before thrombectomy (*n* = 8), distal vessel occlusion (*n* = 30), and reperfusion achieved with direct aspiration alone without a stent retriever (*n* = 37). The final analytic cohort comprised 56 first-line SOLUMBRA procedures with APERIO (Acandis GmbH, Pforzheim, Germany; *n* = 28), Trevo NXT (Stryker Neurovascular, Fremont, CA, USA; *n* = 14) or EmboTrap III (Neuravi Ltd., Galway, Ireland; *n* = 14). Anatomically eligible occlusions included ICA terminus, M1, proximal dominant M2 and basilar-tip occlusions. The single included M2 case involved a proximal dominant M2 branch and was therefore distinguished from excluded distal occlusions. Direct patient identifiers were removed before statistical analysis. Although a complete 90-day follow-up dataset was desirable for secondary clinical outcomes, the exclusion of patients solely because follow-up had not matured may introduce selection bias for the angiographic primary endpoint.

There was no deliberate brand-specific chronological allocation or planned changeover of devices during the study period. For each thrombectomy, the neurointerventional operator selected an anatomically appropriate stent-retriever size from the available inventory. Procedure-level calendar dates were omitted from the de-identified statistical export, preventing assessment of brand-by-calendar-period overlap within that dataset. Temporary shortages of balloon-guide catheters (BGCs) and, at other times, compatible femoral introducer sheaths affected whether balloon-guide support could be used. Such inventory variation may still coincide with calendar time and changes in procedural workflow; temporal confounding cannot be excluded.

**Figure 1:**
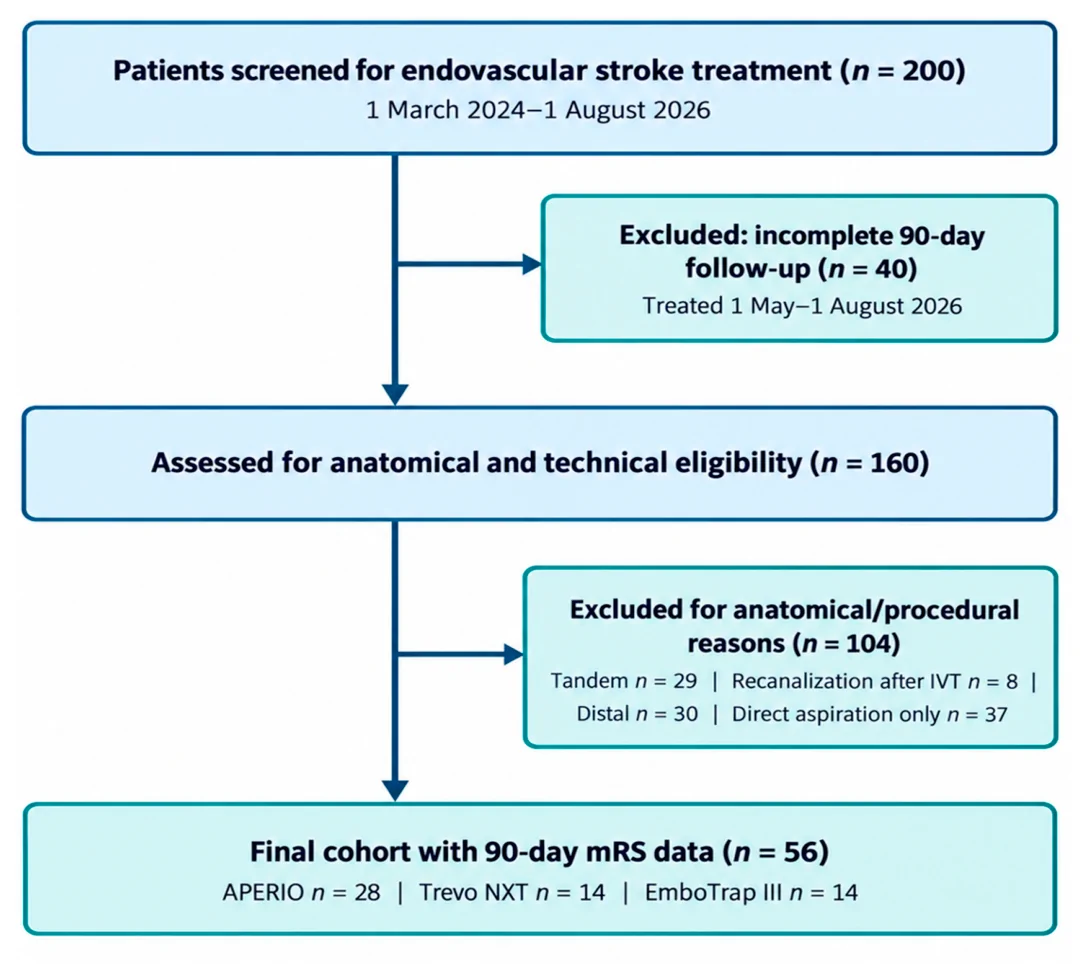
Patient-selection flow. Arrows indicate the progression through screening and eligibility assessment, while box colors are used solely for visual differentiation and have no additional statistical meaning. The exclusion counts were reconstructed by the investigators as non-overlapping primary categories (*n* = 144); prospectively recorded reason-by-reason screening fields were not preserved in the de-identified analytic dataset. Exclusion for incomplete 90-day follow-up may have introduced selection bias.

Figure 1 shows the author-reconstructed screening and exclusion accounting. The five overall exclusion reasons were assigned as non-overlapping primary categories for reporting; this classification is retrospective, not a prospectively recorded screening log. In particular, follow-up maturity was an eligibility criterion despite angiographic strict FPE being the primary outcome.

### 2.2. Endovascular Procedure and Data Collection

All 56 analyzed procedures were performed by one neurointerventional neurologist using a combined stent-retriever-plus-aspiration approach (SOLUMBRA). Aspirating catheters were selected according to anatomy, procedural requirements and inventory: AXS Catalyst 5 (0.058-inch inner diameter; Stryker Neurovascular, Fremont, CA, USA), AXS Catalyst 6 (0.060-inch; Stryker Neurovascular, Fremont, CA, USA), and CEREGLIDE 71 (0.071-inch; Cerenovus, Inc., Miami, FL, USA). The aggregate distribution by first-line retriever was retrospectively confirmed: APERIO—CEREGLIDE 71 *n* = 10, Catalyst 6 *n* = 9, Catalyst 5 *n* = 9; Trevo NXT—*n* = 5, *n* = 5, and *n* = 4, respectively; EmboTrap III—*n* = 7, *n* = 4, and *n* = 3. Patient-level linkage of aspiration catheter model to reperfusion results was unavailable in the analysis dataset, precluding catheter-adjusted models. The first-line stent retriever defined the exposure group, irrespective of later rescue. BGC support was not used when BGCs or compatible femoral introducer sheaths were unavailable. Two device exchanges were verified from procedural records. In case 41, Trevo NXT 6.0 × 37 mm was used for three passes; the device was changed to APERIO 6.0 × 50 mm, and successful reperfusion was obtained on pass four (corrected total passes: four). In case 109, APERIO 4.5 × 50 mm was exchanged for APERIO 6.0 × 50 mm for the second pass. First-pass and final eTICI were both documented as 2b for case 109; these categories were retained, as a second pass and within-grade angiographic differences do not independently justify changing the documented eTICI classification. One rescue exchange was cross-brand and one was within-brand size exchange.

No independent device-selection algorithm was used. Temporary variability in access to BGCs and compatible introducer sheaths, together with nonstandardized aspiration-catheter selection, unavailable patient-level catheter–outcome linkage, and other unmeasured procedural details, means that the exposure contrast represents device-containing thrombectomy strategies rather than the isolated mechanical effect of each retriever.

Baseline variables included age, sex, pre-stroke modified Rankin Scale (mRS), admission National Institutes of Health Stroke Scale (NIHSS), intravenous thrombolysis, onset-to-puncture time, puncture-to-reperfusion time, occlusion site, BGC use, device diameter and length, and total number of thrombectomy passes. Conventional ASPECTS was used for anterior-circulation occlusions. The four basilar-tip occlusions were evaluated using posterior-circulation ASPECTS (pc-ASPECTS) and were excluded from the conventional ASPECTS summary. Safety variables included embolization to new territory, distal embolization, vessel perforation, subarachnoid hemorrhage (SAH), and symptomatic intracranial hemorrhage (sICH). Clinical outcomes included 24 h and discharge NIHSS, 90-day mRS, functional independence (mRS 0–2), and 90-day mortality.

### 2.3. Angiographic Definitions and Outcomes

Angiographic reperfusion was graded using the expanded Treatment in Cerebral Ischemia (eTICI) scale. Modified FPE was defined as first-pass eTICI ≥ 2b, strict FPE as first-pass eTICI 2c–3, and first-pass complete reperfusion as eTICI 3. The primary endpoint was strict FPE. Final angiographic outcomes were final eTICI ≥ 2b, eTICI 2c–3, and eTICI 3. Total pass count was the number of thrombectomy attempts performed to the final procedural state.

Secondary procedural outcomes included modified FPE, first-pass complete reperfusion, final eTICI endpoints, total pass count, puncture-to-reperfusion time, and device crossover/rescue. Safety outcomes included embolization to new territory, distal embolization, vessel perforation, SAH, and intracranial hemorrhage. Clinical outcomes included 24 h and discharge NIHSS, 90-day mRS, functional independence (mRS 0–2), and 90-day mortality.

Fourteen cases had initially been flagged as possible symptomatic intracranial hemorrhage (sICH) in the original source dataset. These 14 records underwent retrospective clinical/radiological re-review using Heidelberg Bleeding Classification criteria [13]. Five were adjudicated as sICH on the basis of hemorrhage-associated neurological deterioration, documented hemorrhage-related intervention and clinical causality assessment; all five showed an admission to 24 h NIHSS increase of 4–10 points and had 90-day mRS 6. The remaining nine initial flags did not meet the final sICH definition. The initially flagged 14 constitute a review subset and should not be confused with all radiologically recorded SAH events in the 56-patient source dataset.

The treating neurointerventional operator assessed and documented the first-pass and final eTICI grades contemporaneously during each procedure; these values were subsequently extracted for retrospective analysis rather than reconstructed from memory. No blinded second reader or independent angiographic core laboratory adjudication was performed, so interobserver agreement (including kappa) could not be estimated. This is particularly important for the eTICI 2b/2c boundary that defines the primary strict FPE endpoint.

### 2.4. Statistical Analysis

Continuous variables were summarized as median and interquartile range (IQR), and categorical variables as counts and percentages. Continuous variables were compared across the three device groups using the Kruskal–Wallis test. Categorical variables were compared using Fisher–Freeman–Halton exact tests because of small expected cell counts. Device-specific strict FPE proportions were reported with exact Clopper–Pearson 95% confidence intervals (CIs). Pairwise exact comparisons for the primary endpoint were adjusted using the Holm method. Secondary and safety endpoints were considered exploratory; to provide multiplicity context, Holm-adjusted *p*-values were additionally calculated across the binary secondary endpoints.

Because device groups were small (28/14/14), Firth penalized logistic regression was used for exploratory adjusted estimation, with APERIO as the reference category. The principal parsimonious model adjusted device group for ICA-T occlusion; this selection reduced parameter count but could not control all relevant confounders. Additional exploratory models individually added admission NIHSS, onset-to-puncture time or BGC use, while sensitivity analyses restricted the cohort to ICA-T/M1 occlusions and to procedures without BGCs. BGC use had no overlap in the Trevo NXT group; neither BGC adjustment nor restriction can fully address confounding. Absolute risk differences were accompanied by Newcombe hybrid-Wilson 95% confidence intervals (CIs), device-specific proportions by exact Clopper–Pearson 95% CIs, and categorical outcomes by Fisher–Freeman–Halton exact tests. Kruskal–Wallis tests were used for continuous variables; the Holm method was used to contextualize multiple testing. Analyses were performed using R statistical software version 4.6.1 (R Foundation for Statistical Computing, Vienna, Austria), including the logistf package for Firth penalized logistic regression. No documented formal a priori sample-size calculation or dated analysis plan was available for the retrospective analysis. Consequently, inferential adjustments and sensitivity analyses are described as exploratory rather than prospectively prespecified. For context, at two-sided α = 0.05 and target power 80%, approximate normal-theory calculations with 28 versus 14 patients and the observed APERIO strict FPE proportion (16/28) indicate detectable absolute differences on the order of +36.5 or −42.1 percentage points. This is a post hoc illustration of sample-size limitations, not an achieved-power claim.

### 2.5. Ethics

The study was conducted in accordance with the Declaration of Helsinki and was approved by the Niğde Ömer Halisdemir University Non-Interventional Clinical Research Ethics Committee (decision no. 2026/209). The requirement for individual informed consent was waived because of the retrospective study design.

## 3. Results

### 3.1. Study Population and Baseline Characteristics

Among 200 patients evaluated for endovascular treatment between 1 March 2024 and 1 August 2026, 40 were excluded because their 90-day follow-up had not been completed at the time of the initial data analysis. Of the remaining 160, 29 were excluded for tandem occlusion, eight for angiographically documented recanalization after intravenous thrombolysis before clot retrieval, 30 for distal vessel occlusion, and 37 for reperfusion with direct aspiration alone. The final cohort comprised 56 first-line SOLUMBRA procedures with a study stent retriever: APERIO *n* = 28, Trevo NXT *n* = 14, and EmboTrap III *n* = 14. All 56 had a recorded 90-day mRS in the analysis dataset. The overall median age was 75.5 years (IQR 61.5–83.3), and 34 patients (60.7%) were women. One Trevo NXT case involved a proximal dominant M2 branch, which was an eligible proximal target under the study’s operational criteria.

Admission NIHSS differed across groups, with median scores of 20.0 (IQR, 18.0–25.3) for APERIO, 18.0 (16.3–20.0) for Trevo NXT, and 25.0 (22.3–27.8) for EmboTrap III (*p* = 0.028). Onset-to-puncture time also differed (240.0 [157.5–300.0], 175.0 [139.5–205.0] and 135.0 [114.0–170.5] min, respectively; *p* = 0.017). BGCs were used in 9/28 APERIO cases (32.1%), 0/14 Trevo NXT cases, and 5/14 EmboTrap III cases (35.7%; *p* = 0.030). The distribution of occlusion sites showed a borderline difference (*p* = 0.053); M1 occlusion was most frequent overall (40/56, 71.4%). Device dimensions reflected platform-specific sizing and were reported descriptively rather than tested as baseline covariates. Aspiration-catheter counts could be reconstructed for each first-line retriever group (Table 1): CEREGLIDE 71 was used in 10 APERIO, 5 Trevo NXT, and 7 EmboTrap III procedures; Catalyst 6 in 9, 5, and 4; and Catalyst 5 in 9, 4, and 3, respectively. These are group-level descriptive distributions, not patient-level catheter-adjusted outcome comparisons.

Conventional ASPECTS values in Table 1 are summarized for anterior-circulation cases only. The four basilar-tip cases (APERIO *n* = 3, Trevo NXT *n* = 1) were graded with pc-ASPECTS (values 8, 8, 9, and 9) and were not pooled into the anterior-circulation ASPECTS summary.

### 3.2. Primary Outcome: Strict First-Pass Effect

**Figure 2:**
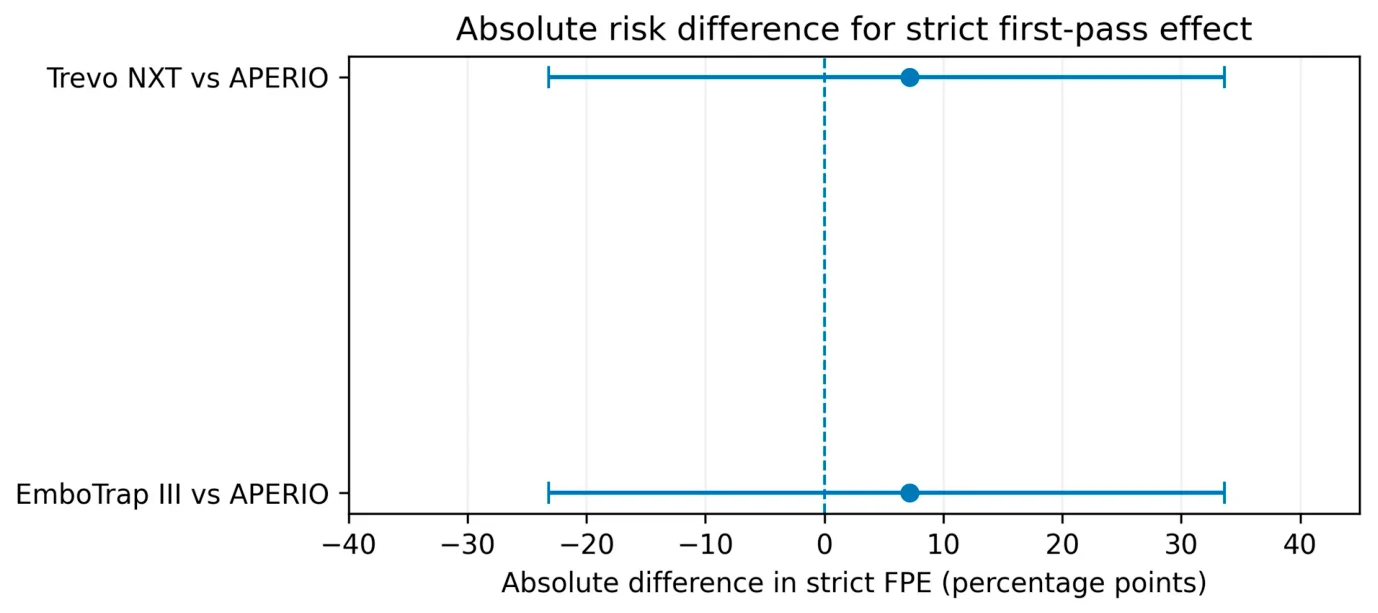
Absolute risk differences in strict first-pass effect relative to APERIO. Points show the absolute percentage-point difference; horizontal bars show Newcombe hybrid-Wilson 95% confidence intervals. Neither comparison excludes clinically important benefit or harm.

Strict FPE occurred in 16/28 APERIO cases (57.1%; exact 95% CI, 37.2–75.5), 9/14 Trevo NXT cases (64.3%; 95% CI, 35.1–87.2), and 9/14 EmboTrap III cases (64.3%; 95% CI, 35.1–87.2). The overall three-group comparison was not statistically significant (Fisher–Freeman–Halton exact *p* = 0.879). Relative to APERIO, the absolute risk difference was +7.1 percentage points for Trevo NXT (Newcombe 95% CI, −23.2 to 33.6) and +7.1 percentage points for EmboTrap III (95% CI, −23.2 to 33.6). Pairwise exact comparisons were non-significant, and all Holm-adjusted *p*-values were 1.000. The primary strict FPE results, together with secondary angiographic, procedural, safety, and clinical outcomes, are summarized in Table 2. The absolute risk differences and their confidence intervals are shown in Figure 2.

The strict FPE row is the primary endpoint; other *p*-values are exploratory, unadjusted global tests. The total-passes *p*-value is recalculated after source correction of case 41 (3 → 4 attempts). Device crossover/rescue includes one cross-brand and one within-brand size exchange. HBC sICH (0/3/2) is the final adjudicated endpoint, distinct from 14 initial possible-sICH source flags. Five subarachnoid hemorrhage cases occurred in the full 56-patient cohort, three of which overlapped with sICH. Holm-adjusted *p*-values for subarachnoid hemorrhage and sICH were 0.109 and 0.485, respectively. Clinical outcome denominators were 28/14/14, all with recorded 90-day mRS. HBC, Heidelberg Bleeding Classification; mRS, modified Rankin Scale; sICH, symptomatic intracranial hemorrhage.

### 3.3. Adjusted Analysis

In the primary Firth penalized logistic regression adjusted for ICA-T occlusion, neither Trevo NXT nor EmboTrap III was independently associated with strict FPE compared with APERIO (Table 3). The adjusted OR was 1.29 for Trevo NXT (95% CI, 0.35–5.09; *p* = 0.707) and 1.29 for EmboTrap III (95% CI, 0.35–4.98; *p* = 0.700). ICA-T occlusion itself was not associated with strict FPE (OR, 1.01; 95% CI, 0.26–4.19; *p* = 0.988). In the NIHSS stress model, device estimates remained non-significant (Trevo NXT OR 1.13, 95% CI 0.29–4.56; EmboTrap III OR 1.45, 95% CI 0.39–5.77). In the onset-to-puncture stress model, the corresponding ORs were 1.27 (95% CI 0.33–5.19) and 1.26 (95% CI 0.32–5.17). The wide confidence intervals reflect substantial uncertainty from the small device groups.

### 3.4. Sensitivity and Stress-Test Analyses

In exploratory analyses restricted to ICA-T/M1 occlusions (*n* = 51), strict FPE was achieved in 14/25 APERIO (56.0%), 7/12 Trevo NXT (58.3%), and 9/14 EmboTrap III (64.3%) cases (global *p* = 0.932). In the no-BGC subset (*n* = 42), the corresponding rates were 11/19 (57.9%), 9/14 (64.3%), and 6/9 (66.7%), respectively (global *p* = 1.000). Firth regression restricted to patients without BGC use and adjusted for ICA-T yielded ORs of 1.22 (95% CI, 0.30–5.15) for Trevo NXT and 1.34 (0.28–6.99) for EmboTrap III versus APERIO. The point estimates were numerically similar across several models, but the wide CIs, sparse cells, and lack of BGC overlap preclude concluding that confounding was removed or that findings were robust.

### 3.5. Secondary Angiographic and Procedural Outcomes

Modified FPE (first-pass eTICI ≥ 2b) occurred in 71.4%, 64.3% and 71.4% of APERIO, Trevo NXT and EmboTrap III cases (global *p* = 0.929). First-pass complete eTICI 3 reperfusion occurred in 46.4%, 57.1% and 50.0%, respectively (*p* = 0.938). Final eTICI ≥ 2b was achieved in 100%, 100% and 92.9% (*p* = 0.500); final eTICI 2c–3 in 75.0%, 92.9% and 85.7% (*p* = 0.441). Final eTICI 3 occurred in 53.6%, 85.7% and 64.3% (*p* = 0.122). Median passes remained one in each group, but after correction of case 41 from three to four passes, the global Kruskal–Wallis *p*-value for pass count was 0.773 (previously 0.785). Two patients underwent rescue device exchange (one cross-brand and one within-brand size exchange). Median puncture-to-reperfusion times were 21.0, 27.5 and 20.0 min, respectively (*p* = 0.077); see Table 2.

### 3.6. Safety and Clinical Outcomes

After Heidelberg re-adjudication of 14 initially flagged possible sICH cases, five of 56 (8.9%) met the final sICH definition (APERIO 0/28, Trevo NXT 3/14 and EmboTrap III 2/14); all experienced hemorrhage-related neurological deterioration and had 90-day mRS 6. The full analytic dataset recorded five SAH events: one with Trevo NXT and four with EmboTrap III, involving the left M1 (*n* = 1), right M1 (*n* = 3), and right ICA terminus (*n* = 1). SAH was detected on follow-up cranial CT. Three of the five SAH events overlapped with PH2 and HBC-adjudicated sICH; these patients required bleeding-related intervention and had 90-day mRS 6. Two SAH events did not meet sICH criteria and had 90-day mRS 3 and 4. In case 185 (EmboTrap III; right M1), perforation occurred during the first pass; after hemorrhage control, thrombectomy was terminated because of vascular fragility, and the patient remained clinically stable. The exploratory exact *p*-values for sICH and SAH were 0.040 and 0.008, respectively; Holm-adjusted *p*-values were 0.485 and 0.109, respectively. No between-device safety inference is justified. The exact SAH onset time and detailed grading of isolated SAH are not recorded in the statistical workbook.

Ninety-day mRS was available for all 56 included cases, with planned assessment at 90 ± 10 days. Surviving patients were assessed during in-person follow-up visits by the treating neurointerventional neurologist, who was aware of device selection; deaths during follow-up were recorded as mRS 6. There was no independent blinded clinical assessment. Mortality was 8/28 (28.6%) for APERIO, 5/14 (35.7%) for Trevo NXT and 4/14 (28.6%) for EmboTrap III (global *p* = 0.929). Ninety-day functional independence (mRS 0–2) was recorded in 18/28 (64.3%), 7/14 (50.0%) and 4/14 (28.6%) cases (*p* = 0.100). In APERIO, mRS 0–2 accounted for 18 patients, mRS 3–5 for two and mRS 6 for eight. Of nine originally flagged hemorrhagic events reclassified as non-sICH, eight deaths were documented as infection-associated multiorgan failure (five pneumonia, three urinary tract infection), with the remaining case surviving at mRS 4. These clinical comparisons are descriptive, not causal evidence of device effectiveness.

## 4. Discussion

This exploratory, single-center cohort yielded strict FPE rates of 57.1%, 64.3%, and 64.3% for APERIO, Trevo NXT, and EmboTrap III. The primary comparison was not statistically significant; however, the absolute between-group estimates were imprecise (risk difference versus APERIO +7.1 percentage points; 95% CI −23.2 to +33.6 for each comparison). These intervals include clinically meaningful advantages and disadvantages. Consequently, the study does not demonstrate equivalence or noninferiority, and it cannot rule out moderate device differences.

Direct comparison with existing device-specific series is limited by differences in first-pass definitions, vascular territories, adjunctive catheter use, and patient selection. Trevo NXT and EmboTrap III studies have reported first-pass near-complete reperfusion under mTICI-based definitions [6,7], whereas APERIO reports have used heterogeneous first-pass and final-reperfusion endpoints [8,9,10]. Accordingly, agreement in point estimates across these published studies should not be interpreted as formal validation of comparable device performance.

BGC use differed substantially across groups: APERIO 9/28 (32.1%), Trevo NXT 0/14, and EmboTrap III 5/14 (35.7%). These differences partly reflected transient shortages of BGCs or compatible femoral introducer sheaths. Although the study did not impose device-brand eras, inventory and procedural practices may still have varied over time. There was no BGC-exposure overlap within the Trevo NXT group. The BGC-adjusted regression and no-BGC restriction therefore provide limited exploratory checks rather than evidence that confounding was eliminated; subgroup comparisons remain vulnerable to selection effects and inadequate precision. The distribution of aspiration-catheter models could be reported for each retriever group; however, catheter inner diameters and procedural positioning were not standardized, and the de-identified workbook did not contain patient-linked catheter models for covariate adjustment. Therefore, the estimates represent comparisons of device-containing SOLUMBRA strategies rather than isolated stent-retriever effects.

Final eTICI ≥ 2b was frequently achieved, but no between-device causal interpretation is justified. Differences in functional outcomes were particularly vulnerable to confounding by the higher admission NIHSS in the EmboTrap III group and by treatment-time differences across groups. Angiographic reperfusion is a procedural surrogate outcome and should not be equated with functional independence or survival. The current cohort was not powered to detect clinically meaningful differences in either 90-day outcome.

Safety observations should be considered descriptive and hypothesis-generating. Five cases met the final Heidelberg sICH criteria after review of 14 initially flagged records, and five SAH events were recorded in the entire source dataset. Three SAH events overlapped with final sICH, while two further SAH events did not; the single recorded perforation occurred in one of these latter cases. Neither sICH nor SAH remained statistically significant after Holm multiplicity correction. Moreover, radiographic outcome adjudication by the treating operator without blinded independent review creates potential classification bias, and the small event counts preclude any attribution of device-specific safety effects.

The study’s informative features are its consistent description of a first-line SOLUMBRA strategy, use of a uniform strict FPE definition, explicit reporting of uncertainty, and transparent characterization of small-cell analyses. The single-operator context provided procedural consistency but cannot be regarded as an unqualified strength because the same operator assessed the primary angiographic outcome without independent blinded adjudication.

This analysis has substantial limitations. First, retrospective availability-driven device selection and differences in NIHSS, onset-to-puncture time, BGC use and occlusion anatomy leave residual and unmeasured confounding. No deliberate brand-specific eras were used, but inventory shortages and procedural practice may still have varied with time; procedure dates were omitted from the de-identified analysis, so temporal balance was not evaluated. Second, small unequal groups (28/14/14) yield wide CIs; illustrative 80%-power calculations suggest detectable risk differences of roughly 36–42 percentage points. Third, follow-up maturity was an eligibility restriction despite an angiographic primary endpoint, increasing selection-bias concerns. Fourth, the treating operator recorded eTICI contemporaneously without blinded second-reader review; the 2b/2c boundary and primary outcome are vulnerable to observer bias. Fifth, retrospective HBC review of hemorrhagic events was nonblinded. Sixth, BGC overlap was absent for Trevo NXT, catheter models were available only as group-level counts rather than patient-linked covariates, catheter positioning was not standardized, and rescue device exchanges were heterogeneous. Seventh, 90-day mRS was assessed nonblinded by the treating operator. These constraints preclude equivalence, noninferiority, causal effectiveness and device-specific safety inferences.

Future multicenter, prospectively designed studies should prespecify inclusion criteria, stratify or standardize proximal balloon support and aspiration-catheter use, capture device allocation by date, record clot and vascular anatomy, and employ blinded angiographic and safety adjudication. Larger cohorts are required to narrow device-specific risk-difference CIs and to establish whether any clinically meaningful efficacy or safety differences exist.

## 5. Conclusions

In this exploratory, single-center retrospective cohort of 56 patients, no statistically detectable difference in strict first-pass reperfusion was identified among APERIO, Trevo NXT, and EmboTrap III. However, the sample was small, confidence intervals were wide, and baseline, temporal and procedural confounding could not be adequately excluded. These preliminary estimates should not be interpreted as evidence of device equivalence, noninferiority, or differential device safety.

## Figures and Tables

**Table 1:** Baseline clinical, imaging, and procedural characteristics by first-line stent retriever.

Characteristic	Overall (*N* = 56)	APERIO (*N* = 28)	Trevo NXT (*N* = 14)	EmboTrap III (*N* = 14)	*p*-Value
Age, years	75.5 (61.5, 83.3)	78.5 (59.8, 86.2)	71.5 (63.2, 77.5)	74.5 (61.8, 77.0)	0.285
Female sex	34 (61%)	18 (64%)	9 (64%)	7 (50%)	0.700
Pre-stroke mRS	1.0 (0.0, 2.0)	1.0 (0.0, 2.0)	1.0 (0.0, 1.0)	1.0 (0.0, 1.0)	0.330
Admission NIHSS	20.0 (18.0, 26.0)	20.0 (18.0, 25.3)	18.0 (16.3, 20.0)	25.0 (22.3, 27.8)	0.028
ASPECTS (anterior circulation only)	9.0 (8.0, 9.0)	8.0 (8.0, 9.0)	9.0 (8.0, 10.0)	9.0 (9.0, 9.0)	0.096
IV thrombolysis	6 (11%)	5 (18%)	0 (0%)	1 (7.1%)	0.300
Onset-to-puncture, min	180.0 (135.0, 242.5)	240.0 (157.5, 300.0)	175.0 (139.5, 205.0)	135.0 (114.0, 170.5)	0.017
Puncture-to-reperfusion, min	23.5 (20.0, 27.0)	21.0 (20.0, 25.0)	27.5 (21.0, 34.2)	20.0 (20.0, 25.8)	0.077
Occlusion site					0.053
M1	40 (71%)	23 (82%)	7 (50%)	10 (71%)	
ICA-T	11 (20%)	2 (7.1%)	5 (36%)	4 (29%)	
M2	1 (1.8%)	0 (0%)	1 (7.1%)	0 (0%)	
Basilar tip	4 (7.1%)	3 (11%)	1 (7.1%)	0 (0%)	
Balloon-guide catheter use	14 (25%)	9 (32%)	0 (0%)	5 (36%)	0.030
Stent-retriever size(s)	—	6.0 × 50: 15; 4.5 × 50: 12; 4.5 × 30: 1	6.0 × 37: 8; 4.0 × 41: 5; 3.0 × 32: 1	6.5 × 45: 11; 5.0 × 37: 3	—
Aspiration catheter model, *n* (%)					
CEREGLIDE 71 (0.071 in)	22 (39.3%)	10 (35.7%)	5 (35.7%)	7 (50.0%)	—
Catalyst 6 (0.060 in)	18 (32.1%)	9 (32.1%)	5 (35.7%)	4 (28.6%)	—
Catalyst 5 (0.058 in)	16 (28.6%)	9 (32.1%)	4 (28.6%)	3 (21.4%)	—

Values are presented as median (interquartile range) or *n* (%). *p*-values are from Kruskal–Wallis or Fisher–Freeman–Halton exact tests, as appropriate. Conventional ASPECTS is reported for anterior-circulation cases; four basilar-tip cases were graded with pc-ASPECTS and are described separately in the text. Catheter inner diameters are manufacturer specifications; catheter-model counts are reported by initial stent-retriever group. Patient-level catheter–outcome linkage was unavailable for adjusted analyses. ASPECTS, Alberta Stroke Program Early CT Score; ICA-T, internal carotid artery terminus; NIHSS, National Institutes of Health Stroke Scale. The single included M2 case was a proximal dominant branch in the Trevo NXT group, distinct from excluded distal vessel occlusions. Baseline denominators are 56 overall unless otherwise indicated; conventional ASPECTS excludes the four posterior-circulation cases.

**Table 2:** Procedural, angiographic, safety, and clinical outcomes.

Outcome	APERIO	Trevo NXT	EmboTrap III	Global *p*-Value
Modified FPE (first-pass eTICI ≥ 2b)	20/28 (71.4%)	9/14 (64.3%)	10/14 (71.4%)	0.929
Strict FPE (first-pass eTICI 2c–3)	16/28 (57.1%)	9/14 (64.3%)	9/14 (64.3%)	0.879
First-pass complete reperfusion (eTICI 3)	13/28 (46.4%)	8/14 (57.1%)	7/14 (50.0%)	0.938
Final eTICI ≥ 2b	28/28 (100.0%)	14/14 (100.0%)	13/14 (92.9%)	0.500
Final eTICI 2c–3	21/28 (75.0%)	13/14 (92.9%)	12/14 (85.7%)	0.441
Final eTICI 3	15/28 (53.6%)	12/14 (85.7%)	9/14 (64.3%)	0.122
Device crossover/rescue	1/28 (3.6%)	1/14 (7.1%)	0/14 (0.0%)	1.000
Embolization to new territory	1/28 (3.6%)	0/14 (0.0%)	1/14 (7.1%)	1.000
Distal embolization	2/28 (7.1%)	0/14 (0.0%)	0/14 (0.0%)	0.491
Vessel perforation	0/28 (0.0%)	0/14 (0.0%)	1/14 (7.1%)	0.500
Subarachnoid hemorrhage	0/28 (0.0%)	1/14 (7.1%)	4/14 (28.6%)	0.008
HBC-adjudicated sICH	0/28 (0.0%)	3/14 (21.4%)	2/14 (14.3%)	0.040
90-day mortality	8/28 (28.6%)	5/14 (35.7%)	4/14 (28.6%)	0.929
90-day mRS 0–2	18/28 (64.3%)	7/14 (50.0%)	4/14 (28.6%)	0.100
Total passes, median (IQR)	1.0 (1.0, 2.0)	1.0 (1.0, 2.0)	1.0 (1.0, 1.0)	0.773
Puncture-to-reperfusion, min, median (IQR)	21.0 (20.0, 25.0)	27.5 (21.0, 34.2)	20.0 (20.0, 25.8)	0.077

**Table 3:** Firth penalized logistic regression for strict first-pass effect across primary and sensitivity analyses.

Analysis	Device Comparison	OR	95% CI	*p*-Value
Unadjusted	Trevo NXT vs. APERIO	1.31	0.37–4.94	0.680
Unadjusted	EmboTrap III vs. APERIO	1.31	0.37–4.94	0.680
Primary adjusted: device + ICA-T	Trevo NXT vs. APERIO	1.29	0.35–5.09	0.707
Primary adjusted: device + ICA-T	EmboTrap III vs. APERIO	1.29	0.35–4.98	0.700
Sensitivity: + BGC	Trevo NXT vs. APERIO	1.23	0.31–5.13	0.767
Sensitivity: + BGC	EmboTrap III vs. APERIO	1.29	0.36–4.94	0.696
Sensitivity: ICA-T/M1 cohort	Trevo NXT vs. APERIO	1.01	0.25–4.28	0.985
Sensitivity: ICA-T/M1 cohort	EmboTrap III vs. APERIO	1.31	0.35–5.12	0.690
Sensitivity: no-BGC cohort	Trevo NXT vs. APERIO	1.22	0.30–5.15	0.784
Sensitivity: no-BGC cohort	EmboTrap III vs. APERIO	1.34	0.28–6.99	0.715
Stress test: + admission NIHSS	Trevo NXT vs. APERIO	1.13	0.29–4.56	0.859
Stress test: + admission NIHSS	EmboTrap III vs. APERIO	1.45	0.39–5.77	0.579
Stress test: + onset-to-puncture	Trevo NXT vs. APERIO	1.27	0.33–5.19	0.730
Stress test: + onset-to-puncture	EmboTrap III vs. APERIO	1.26	0.32–5.17	0.740

APERIO is the reference device in all models. The primary adjusted model included device type and ICA-T occlusion status to limit overfitting and sparse-category instability. BGC use, admission NIHSS, and onset-to-puncture time were assessed in separate sensitivity/stress models. OR, odds ratio; CI, confidence interval; BGC, balloon-guide catheter; ICA-T, internal carotid artery terminus. Primary and stress-test models are exploratory associations, not causal device effects. The no-BGC subgroup comprised 19 APERIO, 14 Trevo NXT, and nine EmboTrap III procedures; treatment-pattern overlap for BGC was incomplete.

## Data Availability

The de-identified data supporting the findings of this study are available on reasonable request from the corresponding author. The data are not publicly available because they originate from confidential patient records; access is subject to institutional and ethics-committee requirements and applicable data-protection regulations.
